# Repeated Stand-Replacing Crown Fires Affect Seed Morphology and Germination in Aleppo pine

**DOI:** 10.3389/fpls.2017.01160

**Published:** 2017-06-30

**Authors:** Antonio Saracino, Alessandro Bellino, Emilia Allevato, Antonio Mingo, Stefano Conti, Sergio Rossi, Giuliano Bonanomi, Domenico Carputo, Stefano Mazzoleni

**Affiliations:** ^1^Department of Agricultural Sciences, University of Naples Federico IIPortici, Italy; ^2^Département des Sciences Fondamentales, Université du Québec à ChicoutimiChicoutimi, QC, Canada; ^3^Key Laboratory of Vegetation Restoration and Management of Degraded Ecosystems, Guangdong Provincial Key Laboratory of Applied Botany, South China Botanical Garden, Chinese Academy of SciencesGuangzhou, China

**Keywords:** fire recurrence, Mediterranean, seed germination, seed morphometry, osmotic potential, *Pinus halepensis*, pH, tree ontogeny

## Abstract

Post-fire reproductive niche of Aleppo pine (*Pinus halepensis*) is deeply interlaced with fire products. Indeed, the high pH and low osmotic potentials of ash beds under burnt crowns constitute the main constraints to seed germination. In this study, we aim to investigate whether fire recurrence, through the physico-chemical constraints imposed by the ash beds, affects the reproduction ability of *P. halepensis* at the germination stage. To this aim, Aleppo pine seeds were collected in neighboring even-aged stands subjected to 0, 1, or 2 fires (namely fire cohorts), and seed morphology and germination performance, in terms of cumulative germination and germination kinetics, were studied under increasing osmotic potentials (from 0.0 to −1.2 MPa) and pH (from 6 to 11). Besides fire history, the role of ontogenetic age of mother plants on seed morphology and germination was also investigated. Differences in seed morphology among the three cohorts have been highlighted in a multivariate context, with anisotropic enlargement of the seeds produced by pine stands experiencing repeated fires. The patterns of seed germination varied primarily in relation to the fire cohort, with seeds from the pine stand experiencing repeated fires exhibiting enhanced tolerance to pH stress. Conversely, germination performances under osmotic constraints mainly depends on tree ontogenetic stage, with an involvement of fire history especially in the timing of seed germination. Our results suggest that, at least in the short term, fire recurrence does not constrain the reproduction ability of Aleppo pine. These results highlight the need for further research to elucidate the mechanisms behind these responses to recurrent fires.

## Introduction

Fire disturbance plays a crucial role in shaping plant communities and selecting plants with fire-related traits in Mediterranean ecosystems. Perennial, non-resprouting species with post-fire seeding ability (obligate seeders *sensu* Pausas and Keeley, 2014) are particularly sensitive to the length of fire return intervals, i.e., the time between two successive fires. These species are particularly affected by fires occurring during the immature stage of their life cycle, before seed reserves have built up, or during senescence, when they are already depleted (Zedler, 1995). Serotiny, the late release of ripe seeds from cones or fruits (Lamont et al., 1991), is a peculiar trait allowing species to maintain a persistent hanging canopy seed bank, usually accessed in hot and dry conditions generated during fire events or by wind (Nathan et al., 1999; Rossi et al., 2017).

Aleppo pine (*Pinus halepensis* Miller) is a perennial species that grows mainly in fire-prone areas along the coast and in the interior of the Mediterranean Basin forming, at landscape level, variable patches of single-cohort stands which can locally disappear when fire is absent over a century (Agee, 1998). The species has a very short juvenile phase (Thanos and Daskalakou, 2000; Climent et al., 2008; Espelta et al., 2008), and builds canopy seed banks (Daskalakou and Thanos, 1996) consisting of both serotinous and non-serotinous cones with anatomically-different scales (Moya et al., 2008), whose ratio is dependent on both environmental and ontogenetic factors (Martín-Sanz et al., 2016). During its life cycle, this species progressively modifies the microenvironment to which its offspring are exposed through pre-fire litter production and necromass retention in the crown, which in turn affect fire intensity and post-fire products (Schwilk and Ackerly, 2001). The physical modification of the surroundings by pine trees is an example of niche construction by inceptive perturbation as labeled by Odling-Smee et al. (2003). The post-fire reproductive niche of Aleppo pine is spatially restricted under the crown projection and radially asymmetric for germination, seedling establishment and growth (Ne'eman, 2000). Ontogenetic modification of the crown shape, from conical to umbrella-like across the life cycle (Shmida et al., 2000) progressively increases the crown surface projection from 20 to 90 m^2^ in trees without crown lateral competition in the old growth stage (Saracino et al., 2002). Low canopy base height (Mitsopoulos and Dimitrakopoulos, 2007, 2014), no self-pruning of dead branches (Keeley and Zedler, 1998; Schwilk and Ackerly, 2001) and no shedding of old open cones (Shmida et al., 2000; Saracino, personal observation) contribute to the propagation of fire from the forest floor up to the crown and an increase in the canopy fuel load. In addition, canopy bulk density actively supports crown fires (Mitsopoulos and Dimitrakopoulos, 2007, 2014). These traits represent a fire-embracing strategy (Schwilk and Ackerly, 2001; Pausas, 2015) aiming to promote the post-fire niche regeneration of Aleppo pine by producing a spatially heterogeneous thickness of ashes (Cerdà and Doerr, 2008).

The intensity of fire affects the quality and quantity of the ashes under the burnt canopy of Aleppo pine trees. Complete plant litter oxidation produces whitish-gray mineral ashes rich in soluble cations (Liodakis et al., 2005), whereas partial oxidation leaves organic components charred, dark and still rich in carbon (Bodí et al., 2014). The ash layer thickness decreases from the stem to the edge of the crown projection, following the radial asymmetry of pre-fire litter accumulation. Increasing ash thickness can inhibit seed germination (Paula et al., 2009) due to the increasing pH and reduction in osmotic potential (Henig-Sever et al., 1996). Moreover, repeated stand-replacing crown fires can reduce growth and reproductive fitness due to a delay in the onset of pine reproduction and a reduction in the number of reproductive pines and cone crop per tree (Eugenio et al., 2006; Espelta et al., 2008). By contrast, Ne'eman et al. (2004) and Goubitz et al. (2004) documented higher levels of serotiny as a positive response to fire.

To sum up, post-fire germination depends on a number of environmental, genetic, and ontogenetic factors which can affect regeneration processes. These factors may be strongly interlaced and it is difficult to assess their role under natural conditions.

In this study we aim to investigate the effect of two main constraints exerted by post-fire substrate conditions, namely osmotic potential and pH, on seed germination both in relation to fire history and ontogenetic age of mother plants. To this end, germination behavior of seeds collected from Aleppo pine cohorts experiencing different fire histories were investigated under simulated conditions of post-fire substrates. Since morphology of the seeds could be involved in germination behavior, a set of morphological traits were also investigated in relation to ontogenetic stage and fire recurrence.

We selected stands located on costal sand dunes in Southern Italy, where the spatially heterogeneous recurrence of summer fires offered a unique opportunity to investigate the role of fire histories under homogeneous environmental conditions.

## Materials and methods

### Study area and seed collection

**Figure 1:**
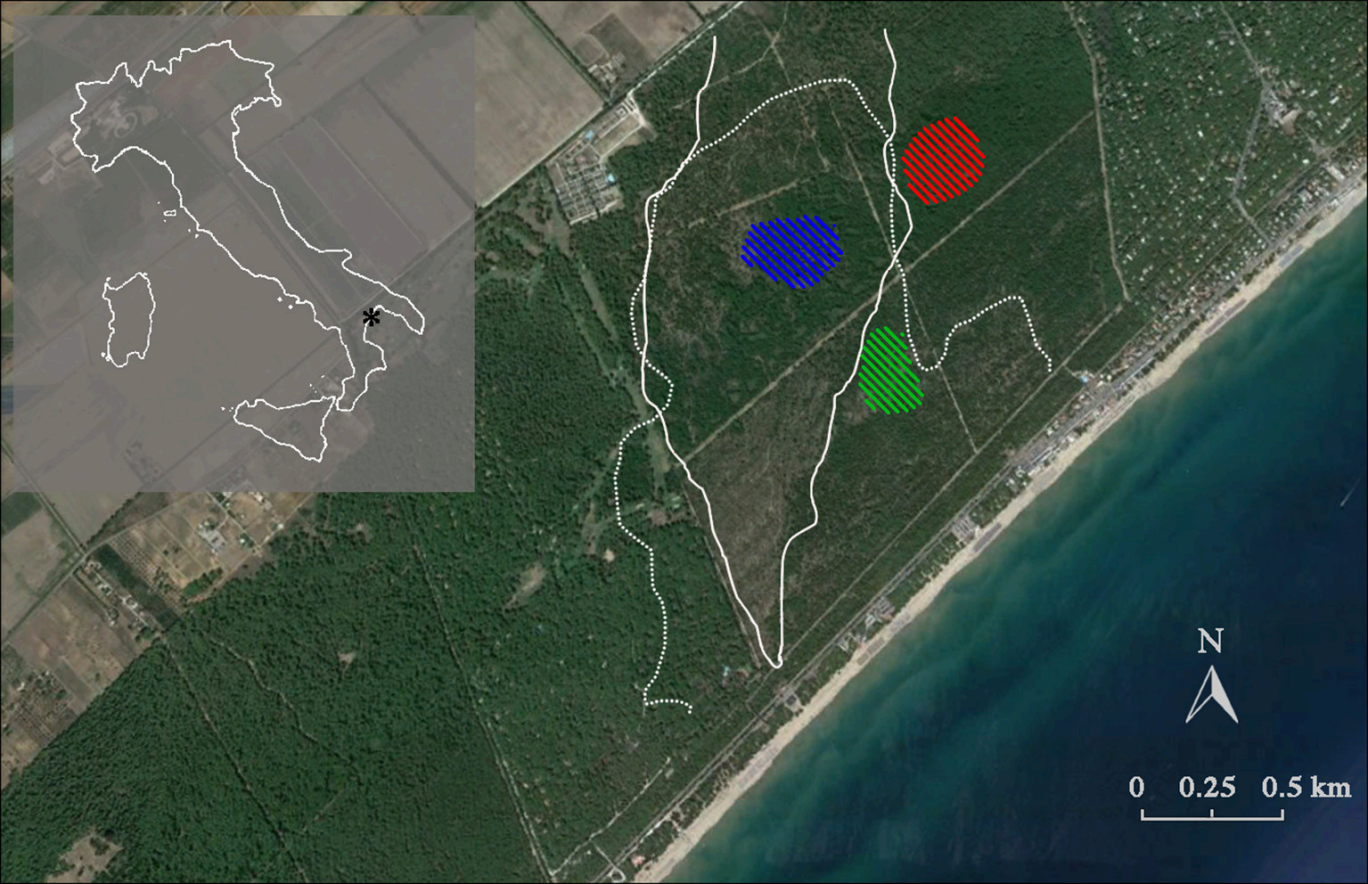
Location of the study site, showing Italy (shaded area) with the relative position of the Biogenetic Natural Reserve Stornara in Apulia region (^*^). The native Aleppo pine (*Pinus halepensis* Miller) forest is registered in the National Book of Seed Forests managed by the Italian Forest Service (MAF, 1960) and is also recognized as a selected seed stand for Aleppo pine of the Mediterranean Basin due to its importance as a germplasm resource (Registration number 52–55, Castellaneta, FAO, 1997). White lines represent the stand-replacing crown fire boundaries (1970: dotted line; 1994: solid line). Diagonal line patterns indicate the tree sampling sites. Red: fire cohort 0 (FC0); green: fire cohort 1 (FC1); blue: fire cohort 2 (FC2).

The study area is located in a native Aleppo pine forest growing on coastal sand dunes in the Biogenetic Natural Reserve Stornara in Apulia region, Southern Italy (40° 27′ 43.46″ N, 16° 55′ 00.59″ E) (Figure 1). The soil is a loose siliceous-calcareous sand (Dumontet et al., 1996). Pine is associated with evergreen sclerophyllous (*Pistacia lentiscus, Phillyrea angustifolia, Myrtus communis, Rhamnus alaternus, Rosmarinus officinalis*), and malacophyllous (*Cistus salvifolius*) shrub species. The climate is semiarid Mediterranean, with a mean annual temperature of 15.8°C, and total precipitation of 535 mm.

Three patches with different fire histories were selected within the same forest, in order to minimize environmental variability and isolate the role of fire histories. Selection was done by using the fire register of the National Forest Service (since 1960) and fire maps drawn up by means of an aerial photogrammetric chronosequence (1947, 1954, 1974, 1990, 2002). In this forest, two stand-replacing crown fires occurred, partially overlapping and forming three patches with different fire history, burning 0, 1, and 2 times (FC0, FC1, and FC2, respectively, Table 1; Figure 1). In FC0, in addition to the analysis of aerial photos, the absence of recent fires was confirmed by the absence of charcoal fragments in the first 40 cm soil profile. In January 2008, 50–100 cones older than 2 years were collected from the whole perimeter of the middle and upper crown of 10 randomly selected plants from each fire cohort. Tree total height and diameter at breast height were measured, using a Tandem clinometer (Suunto, Finland) and a meter tape, respectively, to be used as a proxy of ontogenetic age, for all sampled trees (Table 1). In order to accurately represent the seed pool accessible during a fire event, both brown and serotinous cones were collected.

**Table 1:** Fires and trees characteristics of the three fires cohorts with different fire history, burning 0, 1, and 2 times (FC0, FC1, and FC2, respectively).

**Fire cohorts**	**Fire dates**	**Tree characteristics**		
		**DBH (cm)**	**Height (m)**	**Age (years)**
FC0	–	55.8 ± 10.1	16.4 ± 1.2	85
FC1	July 1970	24.5 ± 2.9	9.0 ± 0.7	37
FC2	July 1970 and August 1994	15.9 ± 4.0	8.4 ± 1.2	13

*Values are given as mean ± s.d. (n = 10 for each fire cohort). DBH represent the diameter at breast height*.

Cones from each plant were separately dried over 1 week in a well-ventilated and shaded environment, put in jute bags and moved to an oven ventilated at 50°C to facilitate scale shrinkage. The seeds extracted were stored in vacuum-type PVC bags at 4°C. From each of the different fire cohorts at least 1200 seeds for the germination tests and 125–150 for morphometric analysis were used. Seeds from the various plants of each fire cohort were pooled together in the germination experiment, in order to investigate the overall germination responses of each cohort. The seeds were carefully selected from those with intact coats. A preliminary cutting test allowed the determination of 11 mg as the threshold mass for fully developed seeds to be used in the experiments. Unwinged seeds were weighed by means of a semi-micro balance to the nearest 10^−2^ mg.

### Morphometric analyses

**Figure 2:**
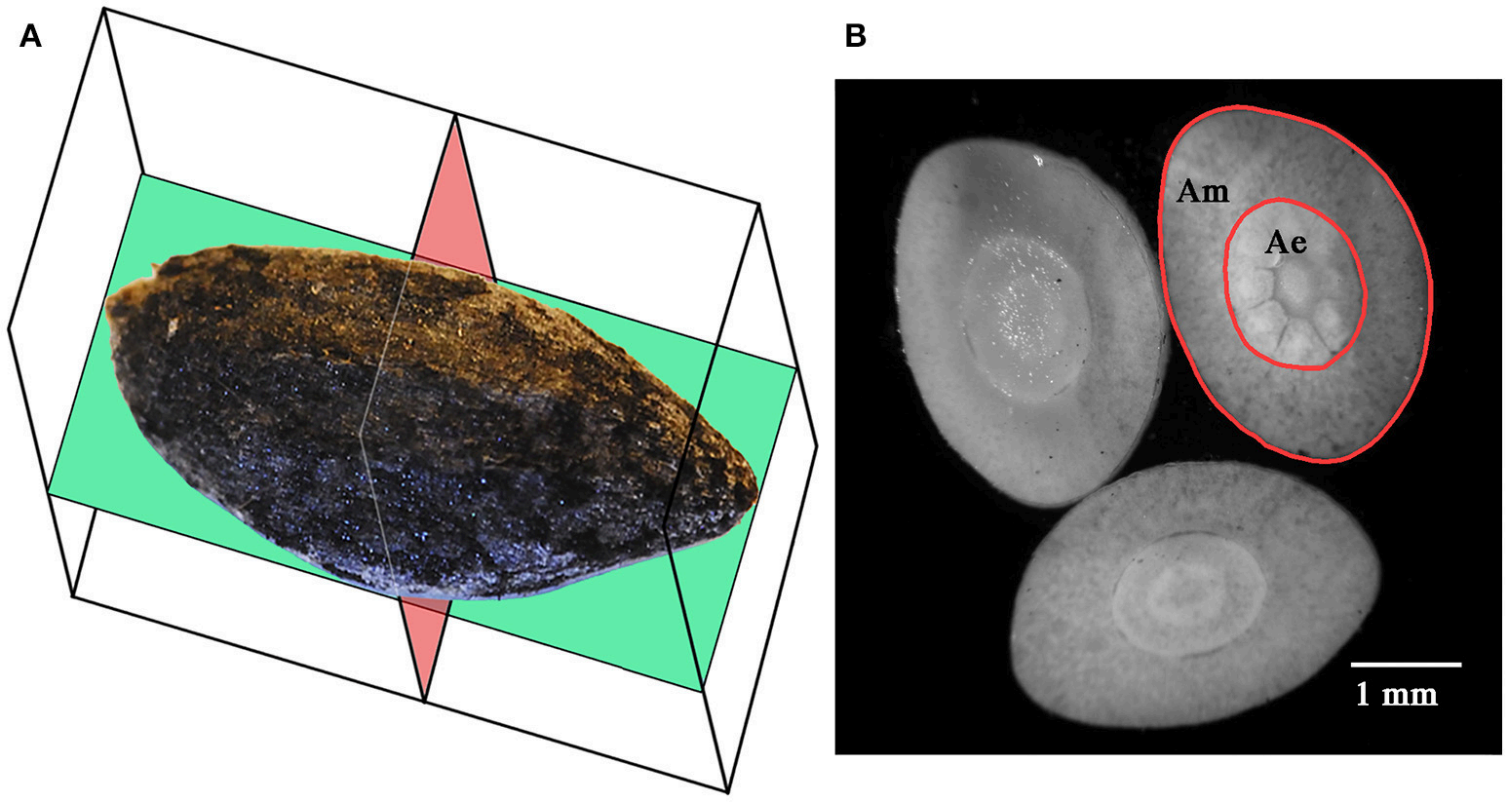
Coronal (green) and axial (red) sectional planes of Aleppo pine seeds **(A)**. Axial sections of an Aleppo pine seed showing the megagametophyte (Am) and the embryo (Ae) **(B)**. Seed in **(A)** is positioned on its adaxial side.

Morphometric analyses were performed on 125–150 seeds per fire cohort (125 from FC0, 125 from FC1, and 150 from FC2), for a total of 400 seeds. Each seed was photographed in its coronal/abaxial plane (Figure 2A; see Kolotelo, 1997 and Terskikh et al., 2005) at 2.5× magnification using an optical microscope (Dialux 20, Leitz, Germany) equipped with a CoolSnap K4 camera (Photometrics, USA). The coronal shape of each seed was then numerically derived from the digital images of the seeds through an *ad hoc* computer script (unpublished data) developed for the R programming environment (R Core Team, 2014), based on the codes reported in Claude (2008). The script (i) imported the binary images of the seeds (threshold performed using the ImageJ 1.43u software package), (ii) derived the Cartesian coordinates of each pixel on the contour of the seed, (iii) calculated the centroid of the contour points, (iv) rotated the point set around the centroid, and (v) compressed the information by storing the length of 36 vectors, from the centroid to the contour points, spread by 10° from each other. The shape of the seeds was then defined by 36 variables, each one corresponding to the length of a radius.

The seeds were carefully uncoated from the micropylar end using hand cutters and weighed again. Uncoated seeds were free-hand cross-sectioned with a razor blade (Figure 2B), obtaining three to five thin sections, which were mounted on a glass slide and photographed with the aforementioned apparatus. The digital images were then analyzed with the free software package ImageJ 1.43u (http://rsb.info.nih.gov/ij/index.html), fitting their perimeters with polylines. The section with the largest surface was selected and used for the measurement of the section area of the embryo with and without the megagametophyte. The area of the megagametophyte and the mass of the coat were calculated as the difference between the previous measurements.

### Germination tests

Germination tests involved experiments under different osmotic potentials (Ψ_π_) and pH. Seeds were germinated in 9 cm disposable Petri dishes over four layers of filter paper wetted with 10 ml of test solution. In order to minimize Ψ_π_ and pH variations over time, seeds were transferred to freshly prepared dishes every 3 days. Petri dishes were placed in the dark in a germination chamber set on a 13 h-15°C:11 h-25°C cycle. This experimental setting aimed to reproduce the environmental conditions experienced by seeds in the field during germination: seeds buried in the dark under 2–3 cm of black post-fire substrate (A. Saracino, unpublished results), at the night:day soil temperatures previously recorded during seedling emergence from October to November (Saracino et al., 1993).

In order to simulate the variable conditions of the post-fire field substrates (Bodí et al., 2014), the germination tests involved six Ψ_π_ levels between 0 and −1.2 MPa, and six pH levels between 6 and 11. Mannitol solutions were employed for the Ψ_π_ germination tests to avoid the toxic effect of polyethylene glycols on the germination of *P. halepensis* seeds (Leshem, 1966), whereas different buffered solutions were employed in the pH germination tests (pH 6.0: MES 50 mM; pH 7.0: MOPS 50 mM; pH 8.0 or pH 9.0: TRIS 50 mM; pH 10.0 or pH 11.0: CAPS 50 mM). Each treatment was replicated five times on batches of 20 seeds. Germinated seeds were counted daily and removed as soon as radicles had protruded until the 28th day after seeding. Ungerminated seeds were then tested for viability by means of tetrazolium tests to refer the germination percentage to the total of viable seeds.

### Data analysis

The overall differences among the fire cohorts in the morphological traits of the seeds were inferred through a multivariate analysis of variance (MANOVA). The mass of the uncoated seeds (Mu), the mass of the coat (Mc), the area of the megagametophyte (Am), the area of the embryo (Ae), and the area of the coronal plane (Ac), were included in the model as dependent variables. A generalized canonical discriminant analysis (CVA) was used to evaluate the contribution of the biometrical traits in differentiating the seeds among the three cohorts. Moreover, a MANOVA and a CVA were performed to analyse the variations in seed shape in relation to the number of fires, using the 36 radii defining the contour of the seeds as dependent variables and the fire cohort as the fixed factor. The relationship between each morphometric parameter and fire history was assessed through linear mixed models using the number of fires and the tree size (both height and diameter at breast height as descriptors of plant ontogenetic stage) as fixed factors and the mother plant as a random factor. The significance of fire history and tree size was then evaluated using a Kenward-Roger adjusted *F*-test.

Cumulative germination percentages (G_*f*_) and mean germination time (MGT), defined as the time when half of the viable seeds had germinated, were selected as descriptors of seed germination performance. G_*f*_ values were logit-transformed, according to Warton and Hui (2011) using the equation:

$$G_{f}=\left(G_{f}+\varepsilon \right)/\left(1-G_{f}+\varepsilon \right)$$

where ε = 0.01 to obtain a normal distribution.

The relation between G_*f*_ or MGT with fire history and tree age was evaluated through ridge regression using the number of fires, tree age and Ψ_π_ or pH as predictors. Tree age, rather than tree size, has been used in analysing germination data, since in the experiments, seeds from various plants were pooled together.

The ridge regression technique was chosen in relation to its L2 regularization, avoiding the shrinkage to zero of the weakest predictors, and to its usefulness in dealing with non-orthogonal problems (Hastie et al., 2009). Moreover, the predictors were scaled to mean zero and unit variance to allow their importance ranking in predicting G_*f*_ and MGT. The penalty parameter (λ) for model selection was chosen using the method proposed by Cule and De Iorio (2012).

The relationship between G_*f*_ or MGT and Ψ_π_ or pH was then modeled separately for the three fire cohorts using linear models and the differences in the slopes were tested with *z*-tests. All analyses and graphics were performed with the stats, lme4, lmtest, nortest, candisc, pbkrtest, ridge, and vioplot packages in R 3.1.1 (R Core Team, 2014).

## Results

**Figure 3:**
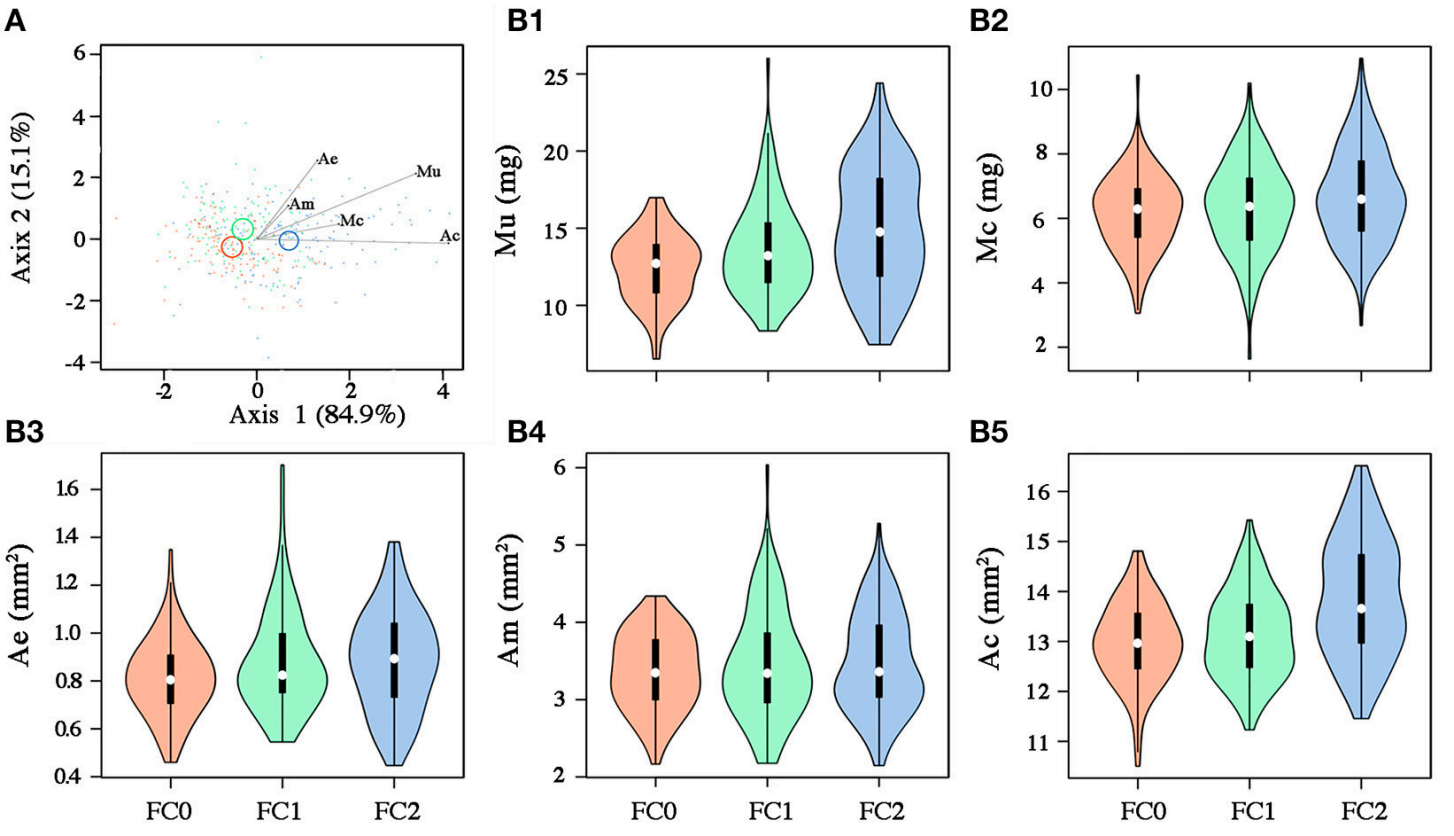
**(A)** Canonical variate analysis on seed morphometric traits (Mu, mass of the uncoated seed; Mc, mass of the coat; Ae, area of the embryo; Am, area of the megagametophyte; Ac, area of the coronal section of the seed). Fire cohorts are coded in different colors (FC0: red; FC1: green; FC2: blue). Vectors indicate the strength and influence of specific morphometric traits on point location relative to canonical axis 1 and axis 2. Confidence circles (for α = 0.05) are also shown, enclosing the true mean of each group at the 95% confidence level and highlighting significant differences among groups in case of no overlap. **(B)** Violin plots for seed morphometric traits: [Mu: **B1**; Mc: **B2**; Ae: **B3**; Am: **B4**; Ac: **B5**]. White circles, black rectangle and upper and lower lines for each violin represent the median, the interquartile range and the upper and lower whisker, respectively. Colored bands represent the kernel density plot.

**Figure 4:**
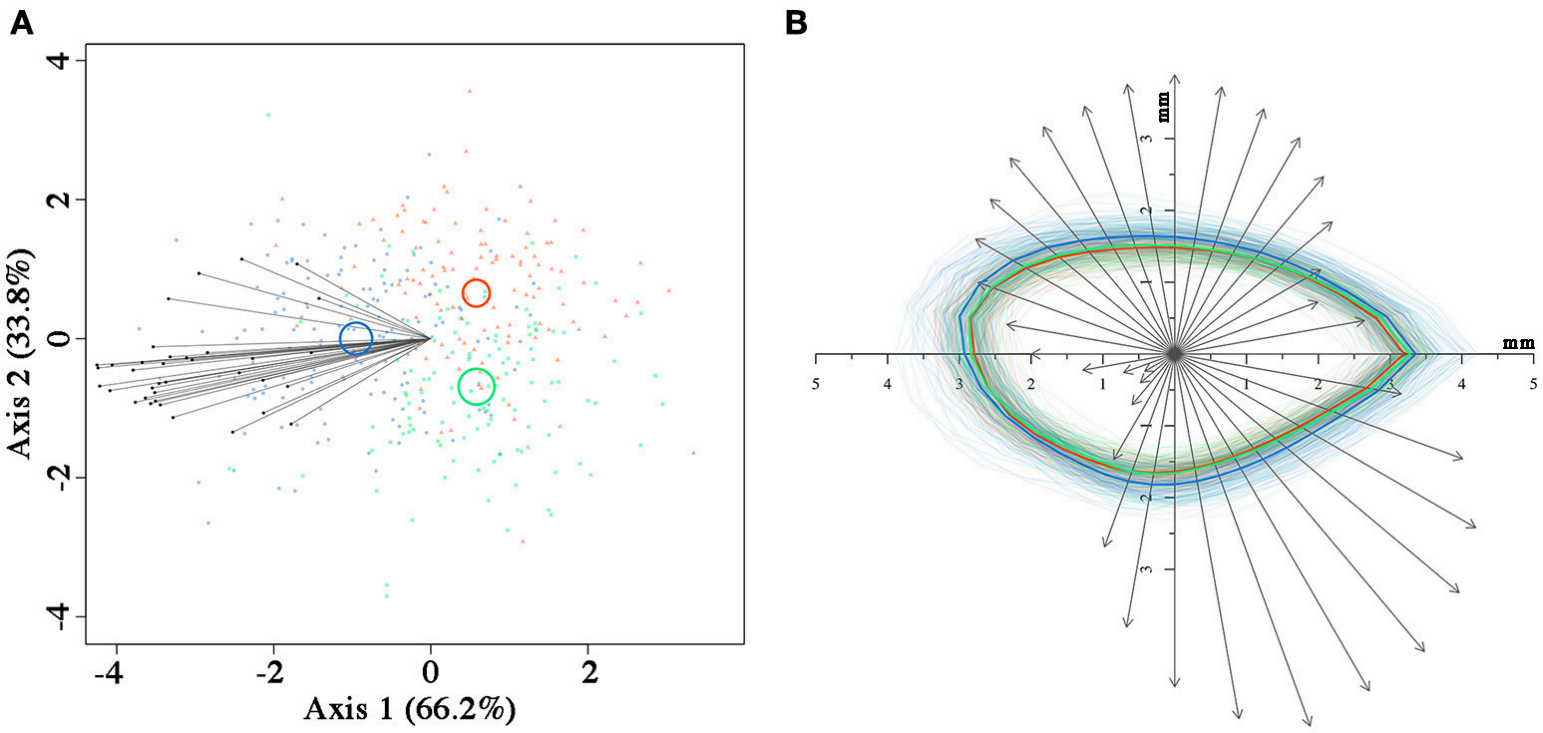
Canonical variate analysis **(A)** on seed coronal shape using the 36 radii length data, with confidence circles (for α = 0.05) highlighted. Superimposition **(B)** of the contour of each seed from the three fire cohorts and of the vectors relative to the first canonical axis from **(A)**. The mean contour for each fire cohort is highlighted with thicker lines. In both **(A,B)**, fire cohorts are coded in different colors (FC0: red; FC1: green; FC2: blue).

**Figure 5:**
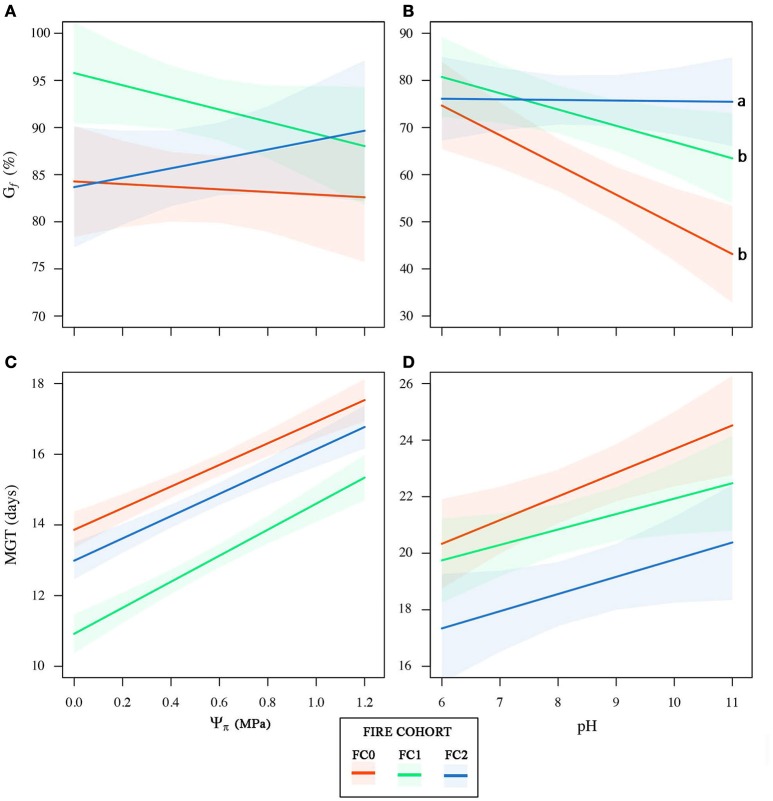
Cumulative germination percentage (G_*f*_) and mean germination time (MGT) as a function of the osmotic potential (Ψπ, **A,C**) and pH **(B,D)** gradients for the three fire cohorts. 95% confidence bands for the linear models for each fire cohort are shown. Letters indicate significant differences between regression slopes according to *z*-test.

Seeds from the three fire cohorts were differentiated in a multivariate context based on their size and mass (Pillai's trace = 0.36, *P* < 0.001), with a clear separation of FC2 seeds from those of the other two cohorts, the confidence circle of which partially overlapped. Morphometric traits differentially contributed to separate the three fire cohorts according to the decreasing order of the vector length: Ac, Mu, Ae, Mc, and Am (Figure 3A). The number of fires was significant in predicting Ac (*P* < 0.05) and Mu (*P* = 0.05), whereas neither the number of fires nor the tree size were significant in predicting the other morphometric traits (Table 2). The variation in Ac and, to a lesser extent, in Mu among the cohorts is highlighted also in Figure 3B, showing the distribution of each morphometric trait in the three fire cohorts through violin plots. Seed shape was significantly affected by the number of fires (Pillai's trace = 0.54, *P* < 0.001) (Figure 4A), with an anisotropic increase in the seed coronal section (Figure 4B). Ridge regression results are reported in Table 3. In the Ψ_π_ experiment, all predictors, namely number of fires, tree size and Ψ_π_, were significant in predicting the mean germination times, with the absolute value of the scaled coefficients (|β|) decreasing in the order tree age > fire number > Ψ_π_. Conversely, tree age had the highest |β| in the cumulative germination model and lower values were associated to Ψ_π_ and then fire number, with *P*-values greater than the 5% confidence limit. Overall, FC1 showed better germination in the Ψ_π_, with higher G_*f*_ and lower MGT than the other two fire cohorts (Figures 5A,C). In the pH experiment, the number of fires had the highest |β| both in the cumulative germination and in the mean germination time models. In the former, tree age |β| was associated with a *P*-value greater than the 5% confidence limit. Furthermore, the number of fires determined an increase in G_*f*_ with a change in the slope in relation to pH, and a monotonic decrease in MGT (Figures 5B,D). Differences in the slopes of the analyzed parameters were observed only in the pH experiment for cumulative germination. Specifically, the slope decreased at increasing fire passages from −0.063 in FC0 to −0.001 in FC2 (Figure 5B). The slopes were significantly (*P* < 0.001 and *P* < 0.05, respectively) different from 0.0 in FC0 and FC1, but not in FC2.

**Table 2:** Summary of the linear mixed models for each morphometric trait (X: Ac, area of the coronal plane; Mu, mass of the uncoated seeds; Mc, mass of the coat; Am, area of the megagametophyte; Ae, area of the embryo) including fire number (left) and tree height (right) as predictors.

		**Fire number**						**Tree height**					
		**Fixed effect**		**Random effect**				**Fixed effect**			**Random effect**		
		**Intercept**	**X**	**σ^2^**	**τ_00,P_**	**ICC_P_**	***R*^2^**	**Intercept**	**X**	**σ^2^**	**τ_00,P_**	**ICC_P_**	***R*^2^**
Ac	B	12,875.67	456.91					14,065.04	−63.57				
	CI	12,400.57	104.07	6.3e+05	328,067.040	0.340	0.448	13,055.28	149.89	635,792.605	413,595.903	0.394	0.448
		13,350.76	809.75					15,074.80	22.75				
	P	<0.001	0.020					<0.001	N.S.				
Mu	B	12.35	1.32					16.14	−0.22				
	CI	10.69	0.09	5.755	4.101	0.416	0.499	12.78	−0.50	5.755	4.634	0.446	0.499
		4.01	2.55					19.50	0.07				
	P	<0.001	0.050					<0.001	N.S.				
Mc	B	6.14	0.27					6.90	−0.04				
	CI	5.48	0.22	1.179	0.623	0.346	0.381	5.64	−0.15	1.179	0.644	0.353	0.381
		6.79	0.75					8.17	0.06				
	P	<0.001	N.S.					<0.001	N.S.				
Am	B	4,194,885.83	91,088.68					4,411,950.47	−10,861.92				
	CI	3,816,414.51	189,999.32	3.48e+11	2.10e+11	0.377	0.405	3,683,511.80	−73,131.5	3.48e+11	2.14e+11	0.382	0.405
		45,73357.14	372,176.68					5,140,389.14	51,407.6				
	P	<0.001	N.S.					<0.001	N.S.				
Ae	B	822,495.60	35,547.96					942,648.35	−7,439.31				
	CI	718,117.20	41,974.54	2.24e+10	1.62e+10	0.419	0.453	743,061.89	−24,501.4	2.24e+10	1.62e+10	0.421	0.453
		926,874.00	113,070.47					1,142,234.80	9,622.8				
	P	<0.001	N.S.					<0.001	N.S.				

*B, coefficient value; CI, 95% confidence interval (upper and lower values represent the lower and upper limit, respectively); P, P-value; σ^2^, within-group (residual) variance; τ_00, P_, between-group-variance; ICC_p_, intraclass correlation coefficient; R^2^, coefficient of determination*.

**Table 3:** Summary of the ridge regression models for cumulative germination percentage (Gf) and mean germination time (MGT) in relation to osmotic potential (Ψ_π_, left) and pH (right) as well as fire number and tree age.

		**Intercept**	**Ψ_π_**	**Fire number**	**Tree age**	**Intercept**	**pH**	**Fire number**	**Tree age**
G_f_	β_ns_	2.432639	−0.056	0.020	−0.004	2.159004	−0.122	0.269	−0.007
	β		−0.208	0.158	−1.140		−2.308	2.491	−2.402
	s.e.m.		0.629	0.424	0.424		1.054	0.900	0.900
	*t*		0.331	0.373	2.691		2.189	2.766	2.668
	P		N.S.	N.S.	<0.01		<0.05	<0.01	<0.01
	λ	0.7559479				0.1389254			
MGT	β_ns_	5.96820	3.228	2.699	0.088	16.34290	0.595	−1.248	0.013
	β		12.078	20.905	24.991		11.214	−11.565	4.274
	s.e.m.		0.974	2.457	2.457		3.178	3.544	3.544
	*t*		12.401	8.509	10.172		3.529	3.263	1.206
	P		<0.001	<0.001	<0.001		<0.001	<0.01	N.S.
	λ	0.02121095				0.08340478			

*β_ns_, non-scaled coefficient value; β, scaled coefficient value; s.e.m., standard error of β; t, t-value associated to β; P, P-value; λ, penalty parameter*.

## Discussion

### Seed morphology

Differences in seed morphology among the three cohorts were highlighted in a multivariate context, with coronal area, the mass of uncoated seed and embryo size as the morphometric traits that contributed more to separate the three fire cohorts. The differentiation among cohorts is primarily related to the coronal area and the mass of the uncoated seeds, indicating an effect of the number of fires on these traits. Although variations in seed coronal area in relation to age/ontogenetic stage of the mother plants could not be definitively excluded, our result strongly suggests that the increase in coronal area, and to a lesser extent in other morphometric traits, is related to the fire history rather than to tree age. Indeed, young pine trees should produce fewer and smaller seeds than older trees in the same environment (Thanos and Daskalakou, 2000), whereas the opposite was observed in our study, supporting the hypothesis that seed size and shape are traits positively affected by fire. Our results could not confirm the influence of fire passages on the size of the megagametophyte and seed coat, which are considered important in the fire ecology of this species (Salvatore et al., 2010). Conversely, embryo size contributed to separate the three fire cohorts, and slightly increased from FC0 to FC2. Such an occurrence can be explained considering the importance of seed germination kinetics on seedling survival, which is a function of the embryo rather than of the megagametophyte. Indeed, larger embryos can germinate earlier (Vivrette, 1995) and overcome intraspecific competition for space, rather than nutrients, which are usually not limiting in post-fire substrates. Improved competitive advantage and seedling survival in larger Aleppo pine seeds, attributed to the higher number of cotyledons, were also reported by Daskalakou and Thanos (2010). Crown ontogeny may instead play a role in determining the higher variability in seed morphological traits exhibited by the younger fire cohort, since we collected cones on both stems and branches.

The variation in seed traits is often involved in plant evolution: in obligate seeders, seed-related traits like shape and mass (Weiher et al., 1999) usually have a major role in both post-fire seedling distribution and establishment (Hammill et al., 1998; Gómez-González et al., 2011, 2016; Shryock et al., 2014; Ruprecht et al., 2015; Jiménez-Alfaro et al., 2016). In our case the observed anisotropic increase in seed size, which leads to a change in seed shape, might affect a number of processes like dispersion, penetration and persistence in post-fire substrates, in which also seed wing traits may be involved. However, we cannot definitively exclude a non-adaptive age dependent plasticity in these traits. Therefore, this topic deserves further investigation.

### Germination patterns

In our study, the patterns of seed germination varied in relation to fire history, with seeds from FC2 exhibiting a greater ability to cope with the constraints imposed by the ash bed in the elective post-fire germination microsite of the crown projection area. Specifically, the number of fires improved seed tolerance to alkaline pH more than to the osmotic potential, both in terms of germination percentage and mean germination time. By contrast, the age of mother plants seems to have a greater effect on the germination performances under osmotic constraints. Cumulative germination showed the clearest responses, gradually shifting from marked inhibition at alkaline pH in FC0 seeds to a neutral response in FC2 seeds. Wood ashes of coniferous species typically reach pH values up to 11 (Raison, 1979; Thomas and Wein, 1990; Henig-Sever et al., 1996), which can inhibit germination. Indeed, Henig-Sever et al. (1996) considered the high soil pH induced by post-fire ash accumulation as the main abiotic factor controlling Aleppo pine forest regeneration, despite the promoting effect of nitrate and ammonium availability on seed germination (Henig-Sever et al., 2000). In addition, leaching by rain is usually not sufficient to produce a favorable seedbed before the occurrence of seed fall, as demonstrated in *P. banksiana* stands (Thomas and Wein, 1994). The neutral response to pH of FC2 seeds suggessts that, at least in the short term, fire recurrence does not constrain the reproduction ability. Actually, it seems that after two repeated stand-replacing crown fires, Aleppo pine has the same, or even a better, capability to overcome the main abiotic constraint for the germination of seeds in post-fire substrates. Total germination under the osmotic constraint is above 80% in all fire cohorts, and age is the most important trait in predicting this behavior. This evidence suggests that fire frequency affects reproductive performance to a lesser extent than osmotic stress and is consistent with previous work which considers this environmental constraint more related to drought than to fire regimes (Falusi et al., 1983; Thanos and Skordilis, 1987). As such, in our case the high resistance to osmotic stress could be considered an adaptation to the semi-arid climate of the area. However, the potential interaction between drought and osmotic potential deserves further investigation, since Aleppo pine seed germination occurs in autumn-winter, when soil is rewetted by precipitation.

The mean germination time pattern in relation to the osmotic potential was non-linear in relation to fire history, with FC1 seeds consistently germinating earlier than the others. This pattern may be related to the length of fire intervals (about 50 y for FC1 vs. 24 y for FC2). Indeed, mother plants of FC1 seeds were recruited on ash beds deeper and richer in soluble cations (Alifragis et al., 2001) than those of FC2 seeds, due to the higher litter and biomass accumulation during the FC1 life span. These features increase both fire intensity and the amount of ash deposited (Cerdà and Doerr, 2008; Bodí et al., 2014), suggesting an involvement of fire also in determining seed resistance to osmotic stresses.

Experimental results on the effects of the osmotic potential on Aleppo pine seed germination vary in relation to seed provenance (Thanos and Daskalakou, 2000). Henig-Sever et al. (1996) pointed out that osmotic potential only marginally affects the germination performance, and Thanos and Skordilis (1987) reported that Aleppo pine can show remarkable germination rates under osmotic stress attaining Ψ_π_ > −1.46 MPa. Conversely, Falusi et al. (1983) showed that Ψ_π_ ranging from −0.2 to −0.6 MPa can reduce germination rates and root growth in some provenances. However, literature results do not resolve the concomitant fire history and seed provenance effects, which limit the reach of the comparisons with our findings. It is well documented that repeated stand-replacing fires reduce the vegetative and reproductive fitness of Aleppo pine by delaying the onset of cone production, reducing the cone crop per tree and the number of reproductive pines (Eugenio et al., 2006; Espelta et al., 2008). In addition, future scenarios in the Mediterranean Basin predict an increase in fire frequency (Mouillot et al., 2002; Pausas and Keeley, 2009) due to a reduction in rainfall (IPCC, 2013). According to our findings, it seems that, at least in the germination phase, fire recurrence does not constrain the reproduction ability of *P. halepensis* due to production of seeds with increased tolerance to the constraints imposed by the ash bed. Instead, the neutral response in relation to pH entails the absence of ash pH constraint on the reproductive niche of the Aleppo pine cohort experiencing repeated fires, ensuring maintenance of fitness and population persistence in fire-prone areas. These changes should occur under several mechanisms implying genetic selection, at the germination stage, of seeds with better germination performance on ash beds. Under the genetic selection hypothesis, the ash layer selective pressure on seed germination would drive population adaptation to repeated fires, with future plants differentially recruited in relation to morphological, physiological or biochemical seed traits, and the eventual fixation of fire-related traits. As known, the environment can also provide adaptive transgenerational plasticity. This is often related to changes to seed provisioning and biochemistry as well as epigenetic mechanisms (reviewed by Herman and Sultan, 2011). They are environmentally sensitive and heritable, and thus play a key role in regulating transgenerational effects following plant stress conditions (Jablonka and Raz, 2009). In this perspective, an alternative explanation of the differences observed among the fire cohorts could be related to short-term adaptation due to epigenetic modifications, e.g., post-translational changes of histone proteins, cytosine DNA methylation, action of small RNA (Danchin et al., 2011). These modifications are heritable, can all cause changes in gene expression and, as recently reported by Yakovlev et al. (2012), may represent an efficient mechanism to survive under changing environments. The age-dependent responses highlighted in this work can be considered positive traits to cope with frequent fire regime. Further studies are required to assess if they are phenotypic traits varying with growth and development, or if they are the result of long-term adaptation of this species to a fire prone environment.

We are confident that this work may open many research fronts, with further research involved in clarifying whether these observed short term responses are able to drive the local adaptation of new ecotypes, eventually allowing the maintenance of the reproduction ability of *P. halepensis* populations under recurrent fire regime.

## Author contributions

AS conceived and coordinated the study. AB participated in the design and performed the statistical analysis with input of SR. AB, AM, EA, and SC carried out experimental work. AS, AB, and EA wrote the main manuscript. All authors contributed to discussing and interpreting the data at all stages.

### Conflict of interest statement

The authors declare that the research was conducted in the absence of any commercial or financial relationships that could be construed as a potential conflict of interest.

## References

[B1] Agee J. K. (1998). Fire and pine ecosystems, in Ecology and Biogeography of Pinus, ed Richardson D. M. (Cambridge: Cambridge University Press), 193–218.

[B2] Alifragis D. Smiris P. Maris F. Kavvadiaa V. Konstantinidou E. Stamou N. (2001). The effect of stand age on the accumulation of nutrients in the aboveground components of an Aleppo pine ecosystem. For. Ecol. Manage. 141, 259–269. 10.1016/S0378-1127(00)00334-0

[B3] Bodí M. B. Martin D. A. Balfour V. N. Santín C. Doerr S. H. Pereira P. (2014). Wildland fire ash: production, composition and eco-hydro-geomorphic effects. Earth Sci. Rev. 130, 130–127. 10.1016/j.earscirev.2013.12.007

[B4] Cerdà J. Doerr S. H. (2008). The effect of ash and needle cover on surface runoff and erosion in the immediate post-fire period. Catena 74, 256–263. 10.1016/j.catena.2008.03.010

[B5] Claude J. (2008). Morphometrics with R. New York, NY: Springer.

[B6] Climent J. Prada M. A. Calama R. Chambel M. R. de Ron D. S. Alía R. (2008). To grow or to seed: ecotypic variation in reproductive allocation and cone production by young female Aleppo pine (*Pinus halepensis*, Pinaceae). Am. J. Bot. 95, 833–842. 10.3732/ajb.2007354 21632409

[B7] Cule E. De Iorio M. (2012). A semi-automatic method to guide the choice of ridge parameter in ridge regression. arXiv:1205.0686v061

[B8] Danchin E. Charmantier A. Champagne F. A. Mesoudi A. Pujol B. Blancet S. (2011). Beyond DNA: integrating inclusive inheritance into an extended theory of evolution. Nat. Rev. Genet. 12, 475–486. 10.1038/nrg3028 21681209

[B9] Daskalakou E. N. Thanos C. (1996). Aleppo pine (*Pinus halepensis*) postfire regeneration: the role of canopy and soil seed banks. Int. J. Wildland Fire 6, 59–66. 10.1071/WF9960059

[B10] Daskalakou E. N. Thanos C. A. (2010). Postfire seedling dynamics and performance in *Pinus halepensis* Mill. populations. Acta Oecol. 36, 446–453. 10.1016/j.actao.2010.05.001

[B11] Dumontet S. Dinel H. Scopa A. Mazzatura A. Saracino A. (1996). Post-fire soil microbial biomass and nutrient content of a pine forest soil from a dunal Mediterranean environment. Soil Biol. Biochem. 28, 1467–1475. 10.1016/S0038-0717(96)00160-5

[B12] Espelta J. M. Verkaik I. Eugenio M. Lloret F. (2008). Recurrent wildfires constrain long-term reproduction ability in *Pinus halepensis* Mill. Int. J. Wildland Fire 17, 579–585. 10.1071/WF07078

[B13] Eugenio M. Verkaik I. Lloret F. Espelta J. M. (2006). Recruitment and growth decline in *Pinus halepensis* populations after recurrent wildfires in Catalonia (NE Iberian Peninsula). For. Ecol. Manage. 231, 47–54. 10.1016/j.foreco.2006.05.007

[B14] Falusi M. Calamassi R. Tocci A. (1983). Sensitivity of seed germination and seedling root growth to moisture stress in four provenances of *Pinus halepensis* Mill. Silvae Genet. 32, 4–9.

[B15] FAO (1997). Directory of Seed Sources of the Mediterranean Conifers. Rome: FAO, Forest Resources Division, Forestry Department [WWW document]. Available online at: http://www.fao.org/DOCREP/006/AD112E/AD112E00.HTM (Accessed 2 October, 2016).

[B16] Gómez-González S. Ojeda F. Torres-Morales P. Palma J. E. (2016). Seed pubescence and shape modulate adaptive responses to fire cues. PLoS ONE 11:e0159655. 10.1371/journal.pone.0159655 27438267 PMC4954725

[B17] Gómez-González S. Torres-Díaz C. Bustos-Schindler C. Gianoli E. (2011). Anthropogenic fire drives the evolution of seed traits. Proc. Natl. Acad. Sci. U.S.A. 108, 18743–18747. 10.1073/pnas.1108863108 22065739 PMC3219139

[B18] Goubitz S. Nathan R. Roitemberg R. Shmida A. Ne'eman G. (2004). Canopy seed bank structure in relation to: fire, tree size and density. Plant Ecol. 173, 191–201. 10.1023/B:VEGE.0000029324.40801.74

[B19] Hammill K. A. Bradstock R. A. Allaway W. G. (1998). Post-fire seed dispersal and species re-establishment in Proteaceous heath. Aust. J. Bot. 46, 407–419. 10.1071/BT96116

[B20] Hastie T. Tibshirani R. Friedman J. (2009). The Elements of Statistical Learning. New York, NY: Springer-Verlag 10.1007/978-0-387-84858-7

[B21] Henig-Sever N. Eshel A. Ne'eman G. (1996). pH and osmotic potential of pine ash as post-fire germination inhibitors. Physiol. Plant. 96, 71–76.

[B22] Henig-Sever N. Eshel A. Ne'eman G. (2000). Regulation of the germination of Aleppo pine *(Pinus halepensis)* by nitrate, ammonium, and gibberellin, and its role in post-fire regeneration. Physiol. Plant. 108, 390–397. 10.1034/j.1399-3054.2000.t01-1-100408.x

[B23] Herman J. H. Sultan S. E. (2011). Adaptive transgenerational plasticity in plants: case studies, mechanisms, and implications for natural populations. Front. Plant Sci. 2:102. 10.3389/fpls.2011.00102 22639624 PMC3355592

[B24] IPCC (2013). Climate Change 2013: The Physical Science Basis. Contribution of Working Groups I to the Fifth Assessment Report of the Intergovernmental Panel on Climate Change. Cambridge, New York: Cambridge University Press.

[B25] Jablonka E. Raz G. (2009). Transgenerational epigenetic inheritance: prevalence, mechanisms, and implications for the study of heredity and evolution. Q. Rev. Biol. 84, 131–176. 10.1086/598822 19606595

[B26] Jiménez-Alfaro B. Silveira F. A. Fidelis A. Poschlod P. Commander L. E. (2016). Seed germination traits can contribute better to plant community ecology. J. Veg. Sci. 27, 637–645 10.1111/jvs.12375

[B27] Keeley J. E. Zedler P. H. (1998). Evolution of life histories in Pinus, in Ecology and Biogeography of Pinus, ed Richardson D. M. (Cambridge: Cambridge University Press), 219–250.

[B28] Kolotelo D. (1997). Anatomy and Morphology of Conifer Tree Seed. Forest Nursery Technical Series 1.1. Victoria: British Columbia, Ministry of Forests, Nursery and Seed Operations Branch.

[B29] Lamont B. B. Le Maitre D. C. Cowling R. M. Enright N. J. (1991). Canopy seed storage in woody plants. Bot. Rev. 57, 277–317. 10.1007/BF02858770

[B30] Leshem B. (1966). Toxic effects of carbowaxes (polyethylen glycols) on *Pinus halepensis* Mill. seedlings. Plant Soil 24, 322–324. 10.1007/BF02232909

[B31] Liodakis S. Katsigiannis G. Kakali G. (2005). Ash properties of some dominant Greek forest species. Thermochim Acta 437, 158–167. 10.1016/j.tca.2005.06.041

[B32] MAF (Ministero dell'Agricoltura e delle Foreste) (1960). Il Libro Nazionale dei Boschi da Seme. Roma, IT: Conifere indigene, MAF, Collana verde 5.

[B33] Martín-Sanz R. C. Santos-del-Blanco L. Notivol E. Chambel M. R. San-Martín R. Climent J. (2016). Disentangling plasticity of serotiny, a key adaptive trait in a Mediterranean conifer. Am. J. Bot. 103, 1582–1591. 10.3732/ajb.1600199 27620182

[B34] Mitsopoulos I. D. Dimitrakopoulos A. P. (2007). Canopy fuel characteristics and potential crown fire behavior in Aleppo pine (*Pinus halepensis* Mill.) forests. Ann. For. Sci. 64, 287–299. 10.1051/forest:2007006

[B35] Mitsopoulos I. D. Dimitrakopoulos A. P. (2014). Estimation of canopy fuel characteristics of Aleppo pine (*Pinus halepensis* Mill.) forests in Greece based on common stand parameters. Eur. J. For. Res. 133, 73–79. 10.1007/s10342-013-0740-z

[B36] Mouillot F. Rambal S. Joffre R. (2002). Simulating climate change impacts on fire frequency and vegetation dynamics in a Mediterranean-type ecosystem. Glob. Chang. Biol. 8, 423–437. 10.1046/j.1365-2486.2002.00494.x

[B37] Moya D. Saracino A. Salvatore R. Lovreglio R. de Las Heras J. Leone V. (2008). Anatomic basis and insulation of serotinous cones in *Pinus halepensis* Mill. Trees 22, 511–519. 10.1007/s00468-008-0211-1

[B38] Nathan R. Safriel U. N. Noy-Meir I. Schiller G. (1999). Seed release without fire in *Pinus halepensis*, a Mediterranean serotinous wind-dispersed tree. J. Ecol. 87, 659–669. 10.1046/j.1365-2745.1999.00382.x

[B39] Ne'eman G. (2000). The effect of burned pine trees on post-fire regeneration, in Ecology, Biogeography and Management of Pinus Halepensis and P. brutia Mediterranean pine Forest Ecosystems, eds Ne'eman G. Trabaud L. (Leiden: Buckhuys Publishers), 303–319.

[B40] Ne'eman G. Goubitz S. Nathan R. (2004). Reproductive traits of *Pinus halepensis* in the light of fire–a critical review. Plant Ecol. 171, 69–79. 10.1023/B:VEGE.0000029380.04821.99

[B41] Odling-Smee F. J. Laland K. N. Feldman M. W. (2003). Niche Construction: The Neglected Process in Evolution (Monographs in Population Biology, 37). Princeton, NJ: Princeton University Press.

[B42] Paula S. Arianoutsou M. Kazanis D. Tavsanoglu Ç. Lloret F. Buhk C. (2009). Fire-related traits for plant species of the Mediterranean Basin. Ecology 90:1420 10.1890/08-1309.1

[B43] Pausas J. G. (2015). Evolutionary fire ecology: lessons learned from pines. Trends Plant Sci. 20, 318–324. 10.1016/j.tplants.2015.03.001 25814325

[B44] Pausas J. G. Keeley J. E. (2009). A burning story: the role of fire in the history of life. BioScience 59, 593–601. 10.1525/bio.2009.59.7.10

[B45] Pausas J. G. Keeley J. E. (2014). Evolutionary ecology of resprouting and seeding in fire-prone ecosystems. New Phytol. 204, 55–65. 10.1111/nph.12921 25298997

[B46] R Core Team (2014). R: A Language and Environment for Statistical Computing. R Foundation for Statistical Computing. Vienna: R Foundation for Statistical Computing.

[B47] Raison R. J. (1979). Modification of the soil environment by vegetation fires, with particular reference to nitrogen transformations: a review. Plant Soil 51, 73–108. 10.1007/BF02205929

[B48] Rossi S. Morin H. Gionest F. Laprise D. (2017). Inter- and intra-annual patterns of seed rain in the black spruce stands of Quebec, Canada. iForest, 10, 189–195. 10.3832/ifor2145-009

[B49] Ruprecht E. Fenesi A. Fodor E. I. Kuhn T. Tökölyi J. (2015). Shape determines fire tolerance of seeds in temperate grasslands that are not prone to fire. Persp. Pl. Ecol. Evol. Syst. 17, 397–404. 10.1016/j.ppees.2015.07.001

[B50] Salvatore R. Moya D. Pulido L. Lovreglio R. López-Serrano F. R. De las Heras J. (2010). Morphological and anatomical differences in Aleppo pine seeds from serotinous and non-serotinous cones. New For. 39, 329–341. 10.1007/s11056-009-9174-3

[B51] Saracino A. D'Alessandro C. M. Maiullari G. Leone V. (2002). Pattern of resin dripping under Aleppo pines (*Pinus halepensis* Mill.) of different crown size, in Fire and Biological Processes, eds Trabaud L. Prodon R. (Leiden: Backhuys Publishers), 291–302.

[B52] Saracino A. Leone V. De Natale F. (1993). Permanent plots for the study of natural regeneration after fire of *Pinus halepensis* Miller in dunal environment. Annali di Botanica (Roma) 51, 209–217.

[B53] Schwilk D. W. Ackerly D. D. (2001). Flammability and serotiny as strategies: correlated evolution in pines. Oikos 94, 326–336. 10.1034/j.1600-0706.2001.940213.x

[B54] Shmida A. Lev-Yadun S. Goubitz S. Ne'eman G. (2000). Sexual allocation and gender segregation in *Pinus halepensis, P. brutia* and *P. pinea*, in Ecology, Biogeography and Management of Pinus halepensis and P. brutia Mediterranean Pine Forest Ecosystems, eds Ne'eman G. Trabaud L. (Leiden: Backhuys Publishers), 91–104.

[B55] Shryock D. F. De Falco L. A. Esque T. C. (2014). Life-history traits predict perennial species response to fire in a desert ecosystem. Ecol. Evol. 4, 3046–3059. 10.1002/ece3.1159 25247062 PMC4161178

[B56] Terskikh V. V. Feurtado J. A. Ren C. Abrams S. R. Kermode A. R. (2005). Water uptake and oil distribution during imbibition of seeds of western white pine (*Pinus monticola* Dougl. ex D. Don) monitored *in vivo* using magnetic resonance imaging. Planta 221, 17–27. 10.1007/s00425-004-1426-z 15605241

[B57] Thanos C. A. Daskalakou E. N. (2000). Reproduction in *Pinus halepensis* and *P. brutia*, in Ecology, Biogeography and Management of Pinus Halepensis and P. brutia Mediterranean Pine Forest Ecosystems, eds Ne'eman G. Trabaud L. (Leiden: Backhuys Publishers), 79–90.

[B58] Thanos C. A. Skordilis A. (1987). The effects of light, temperature and osmotic stress on the germination of *Pinus halepensis* and *P. brutia* seeds. Seed Sci. Technol. 15, 163–174.

[B59] Thomas P. A. Wein R. W. (1990). Jack pine establishment on ash from wood and organic soil. Can. J. For. Res. 20, 1926–1932. 10.1139/x90-258

[B60] Thomas P. A. Wein R. W. (1994). Amelioration of wood ash toxicity and jack pine establishment. Can. J. For. Res. 24, 748–755. 10.1139/x94-099

[B61] Vivrette N. J. (1995). Distribution and ecological significance of seed-embryo types in Mediterranean climates in California, Chile, and Australia, in Ecology and Biogeography of Mediterranean Ecosystems in Chile, California, and Australia (Ecological studies 108), eds Kalin Arroyo M. T. Zedler P. H. Fox M. D. (New York, NY: Springer Verlag), 274–288. 10.1007/978-1-4612-2490-7_11

[B62] Warton D. I. Hui F. K. (2011). The arcsine is asinine: the analysis of proportions in ecology. Ecology 92, 3–10. 10.1890/10-0340.1 21560670

[B63] Weiher E. Werf A. Thompson K. Roderick M. Garnier E. Eriksson O. (1999). Challenging Theophrastus: a common core list of plant traits for functional ecology. J. Veg. Sci. 10, 609–620. 10.2307/3237076

[B64] Yakovlev I. Fossdal C. G. Skrøppa T. Olsen J. E. Jahren A. H. Johnsen Ø. (2012). An adaptive epigenetic memory in conifers with important implications for seed production. Seed Sci. Res. 22, 63–76. 10.1017/S0960258511000535

[B65] Zedler P. H. (1995). Fire frequency in Southern California shrublands: biological effects and management options, in Brushfires in California Wildlands: Ecology and Resource Management, eds Keeley J. E. Scott T. (Fairfield, WA: International Association of Wildland Fire), 101–111.

